# Prevalence of Hepatitis B Virus infection in the Gulf Cooperation Council: a systematic review and meta-analysis

**DOI:** 10.1186/s12879-022-07806-4

**Published:** 2022-11-07

**Authors:** Ali A. Alali, Mahmoud N. Abo-Shehada

**Affiliations:** 1 Faculty of Epidemiology and Population Health, London School of Hygiene and Tropical Medicine, University of London, London, UK; 2 Department of Medicine, Faculty of Medicine, Kuwait University, Jabriyah, Kuwait

**Keywords:** Hepatitis B virus, Prevalence, Systematic review, Gulf Cooperation Council, Arabian Gulf

## Abstract

**Background:**

Hepatitis B virus (HBV) infection is a global public-health problem. Since the introduction of an effective vaccine, the epidemiology of HBV infection is changing. We aimed to estimate the prevalence of HBV infection in the Gulf Cooperation Council (GCC) region and delineate any variation in member-countries, special sub-groups, and over time.

**Methods:**

This is a systematic review and meta-analysis to review studies of HBV prevalence in the GCC region. Databases were searched and all studies from inception to July 31st, 2021, were considered for inclusion. The pooled HBV prevalence was analyzed using the random-effect model after assessment for heterogeneity. True prevalence was adjusted using the Rogan-Gladen estimator. Pre-defined subgroup analysis was performed, and publication bias was assessed.

**Results:**

Overall, 99 studies (n = 1,944,200 participants) met the inclusion criteria. The overall HBV apparent prevalence was 3.05% (95% CI 2.60, 3.52) and the true prevalence was 1.67% (95% CI 1.66, 1.68). The apparent prevalence varied between subgroups. Over time, the apparent prevalence of HBV infection has declined from 9.38% (95% CI 7.26, 11.74) before 1990 to 1.56% (95% CI 1.07, 2.12) during the period 2010 to 2020.

**Conclusion:**

Over the last four decades the overall prevalence of HBV infection in the GCC region has decreased from high- to low-endemicity level. However, due to poor methodology of the included studies, further high-quality community-based studies are needed to obtain more precise estimate of HBV infection in this region.

**Supplementary Information:**

The online version contains supplementary material available at 10.1186/s12879-022-07806-4.

## Introduction

Hepatitis B Virus (HBV) infection is a global health problem with an estimated 2 billion people worldwide exposed to the virus [1]. In 2019, the World Health Organization (WHO) estimated that approximately 296 million people worldwide are chronically infected by HBV making it one of the most common chronic infections on a global scale [2]. Patients with HBV infection have been shown to exert more health and economic burden on society compared to other patients [3, 4]. Despite the high morbidity and mortality associated with HBV infection, it remains a preventable disease. A DNA recombinant vaccine against HBV was approved and introduced into clinical practice in 1986 [5]. It was shown to be effective at reducing the risk of chronic HBV infection and subsequently reducing the risk of HBV-related complications [6].

The Gulf Council Cooperation (GCC) is a regional, intergovernmental, political and economic union located in the Middle East next to the Arabian gulf [7]. This union consists of 6 neighboring countries namely Kingdom of Saudi Arabia (KSA), Kuwait, United Arab Emirates (UAE), Bahrain, Oman and Qatar with an estimated combined population of 54 million people. These countries share many social, economic and cultural properties which makes it a distinctive union. Furthermore, these countries are unique in the Middle East region in being high-income countries with high Human Development Index hence sharing many of the properties of developed countries [8]. This may suggest that the HBV burden in this area is similar to developed countries. On the other hand, half of the people living in this region are non-nationals that come from areas of high HBV prevalence such as Africa and Asia. This matter is complicated further by the policies in the GCC that limits non-nationals from obtaining residency visa in case of HBV infection. Furthermore, universal childhood vaccination and mass vaccination programs against HBV likely resulted in lowering HBV prevalence in the GCC [9]. Although, there is no estimate of the overall HBV prevalence in the GCC region, the WHO estimates that 3.3% of the general population living in the Eastern Mediterranean region (EMR) are infected with HBV [10]. On the other hand, older studies from the GCC countries suggest that HBV infection may be hyperendemic in certain parts of the area [11].

Accurate estimate of the true prevalence of HBV infection in the GCC region is important to measure the burden of the infection and the outcomes of the practiced control measures. This ensures adequate resources are allocated by governments to tackle this public-health problem and achieve HBV elimination by 2030 as endorsed by Global Health Sector Strategy (GHSS) [2]. This systematic review and meta-analysis aims to review relevant studies of HBV infection prevalence in the GCC area, in order to; (1) estimate an overall prevalence of HBV for the GCC region and each of the member countries, (2) assess the change in the prevalence of HBV infection in the GCC over time, (3) estimate the prevalence of some population subgroups and risk groups in the region.

## Methods

A systematic literature review and meta-analysis were conducted employing a predefined protocol based on Cochrane[12] and adhered to the reporting guidelines recommended by the Preferred Reporting Items for Systematic Review and Meta-analysis (Additional file 1: PRISMA) [13].

### Search strategy, selection criteria and quality assessment

The research question was defined using PIOST. The inclusion criteria included observational studies regardless of the design (cross-sectional, cohort and case–control), published in peer-reviewed literature, and grey literature in any language up to July 2021, studied people of both gender, children and adults (older than 18 years), living in the GCC region during the study period and in all settings (healthcare and community-based studies). The status of HBV infection was assessed using a validated test to detect hepatitis B surface antigen (HBsAg), including Reverse Phase Passive Hemagglutination, Enzymes-linked Immunosorbent Assay (ELISA), Chemiluminescent Immunoassays, Chemiluminescent Microparticle Immunoassays (CMIA) and Radioimmunoassay.

The literature search used Medline, EMBASE and Global health databases, and both thesaurus searching (using MeSH) and free-text searching. Search options such as truncations and wild cards were utilized. Boolean operators and the subject headings comparable to the search terms were used when available. In addition, the reference list of reviewed articles were screened for any available references. EndNote™ (version 20.1) software was used as the reference manager for the study. The details of the search strategy are shown in Additional file 2: Appendix A1.

The retrieved study titles were screened, and the study was excluded if it did not relate to the study topic or duplicate. The abstracts of the remaining studies were screened and excluded if irrelevant. The full texts of the remaining studies were retrieved and their references searched for eligible articles. For the articles that were not immediately available, the authors and/or the journal were contacted to obtain a copy of the study. After full text assessment, studies that fulfilled the inclusion criteria were included in this study. The study screening and study selection was performed by the first author only.

### Quality assessment

Quality assessment of the included studies was performed using a standard tool (Additional file 2: Appendix B) that consists of criteria adapted from Cochrane guidelines [12] and Downs & Black guidelines for cross-sectional studies [14]. The tool assigned a score for different domains and a total score was calculated. Different tools were available for different study designs (cross-sectional, cohort and case–control). The assessment tool for cross-sectional studies measured the following: representativeness of the sample to the target population, measurement of the exposure using a valid tool, recognizing and addressing main potential confounders, utilizing and descripting appropriate statistical tools, addressing missing data, reporting confidence intervals, and estimating random variability in the data for the main outcome. For cohort and case–control studies, the tool measured similar variables with slight variation. The studies were categorized into “low”- and “high” quality based on the quality assessment tool using a cutoff of ≥ 5 points for cross-sectional studies, ≥ 6 points for case–control studies and ≥ 7 points for cohort studies with higher score corresponding to higher quality.

### Extracting relevant data

Data extraction of the included studies was done systematically using data extraction forms and data were entered into database. The study population was categorized according to the perceived risk of acquiring HBV infection to, *high-risk group* that includes hemodialysis patients, Persons Who Inject Drugs (PWID), people with liver disease, patients who required repeated blood transfusion such as hereditary hemolytic anemia (e.g. sickle cell anemia and thalassemia) and congenital coagulopathy, and *average-risk group* that represents the general population, pregnant women, blood donors, army recruits, healthy adults/children, controls from case–control studies and health-care workers. The decision to include health-care workers in this category is related to the fact that despite their risk of exposure to HBV at work, they are usually fully vaccinated and they apply prophylactic measures of HBV transmission. This system was adopted with modification from a previously published study[15].

### Analysis strategy

Meta-analyses were performed using STATA (version 15.1). Estimates were pooled using a DerSimonian-Laird random effect model which assumes that the true effect size could vary from study to study, and the true effects are normally distributed [16]. The magnitude of variation between studies due to heterogeneity was quantified using the Higgin’s I^2^ statistics and Cochran’s Q test. An I^2^ statistics was interpreted as percentages with values of 25%, 50% and 75% approximately corresponding low, moderate and high between-study heterogeneity [17]. A Cochran’s Q test *p*-value < 0.1 was consistent with significant heterogeneity. The fixed-effect model was used when heterogeneity was low, whereas the random-effect model was used when heterogeneity was high. In addition, a sensitivity analysis was performed after excluding studies with low quality measurement. Forrest plots with description of the findings were generated to present the results and calculate the point estimate and the 95% CI. Publication bias was assessed using Egger’s test and funnel plot. The pooled apparent prevalence was adjusted using the sensitivity and specificity of the used test to calculate the true prevalence according to Rogan-Gladen [18]. The true prevalence for studies that used ELISA [sensitivity 98% and specificity 97%] [19] and CMIA [sensitivity 88.9% and specificity 98.9%] [20] were calculated using the reported sensitivity and specificity of the test used and the pooled estimate from the meta-analysis.

## Results

### Literature search

**Fig. 1:**
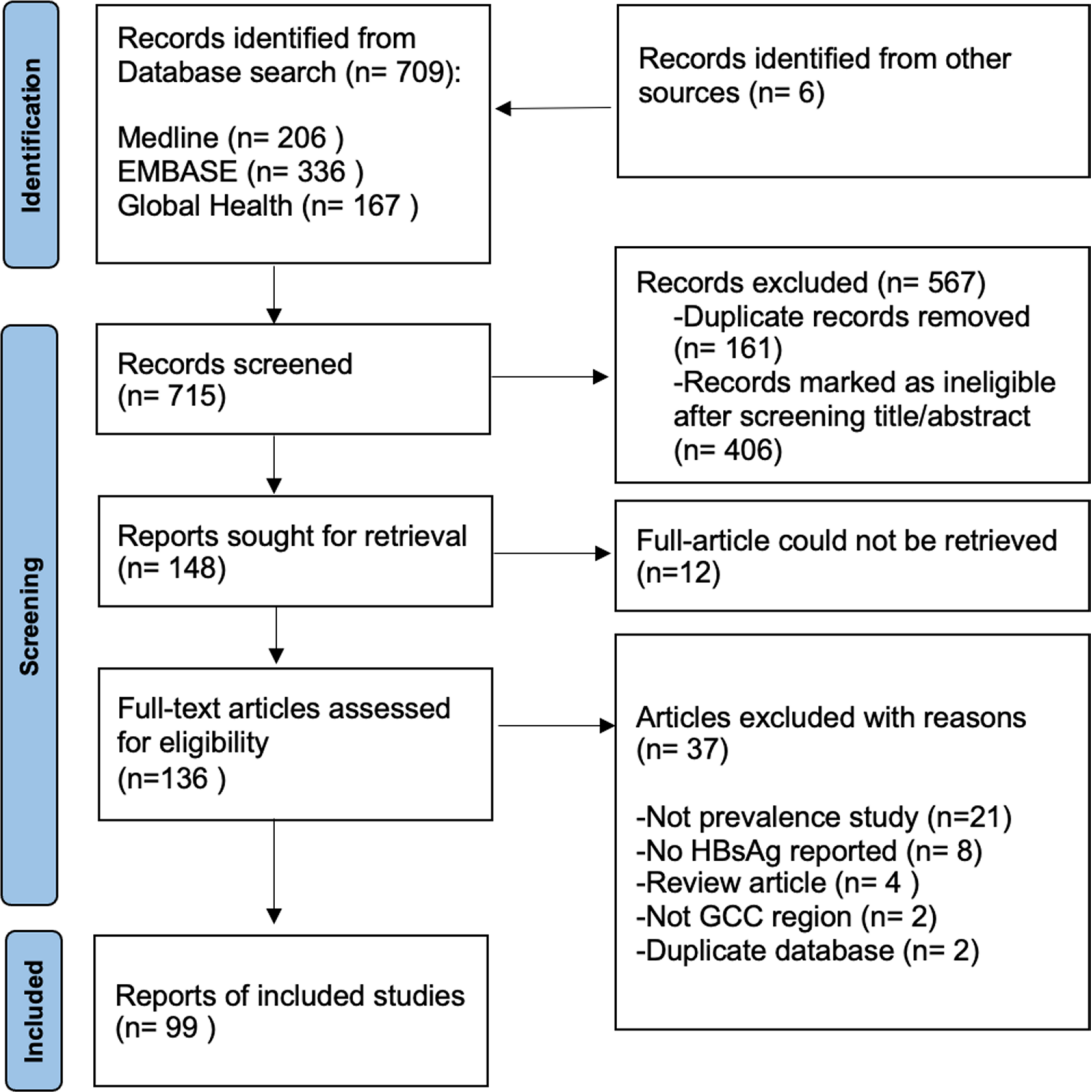
PRISMA flow diagram showing the number of articles identified, screened and included

The flow diagram (Fig. 1) shows the number of articles identified, screened, and included in the systematic review. A total of 709 records were identified from the three searched databases. After removal of duplicates, the remaining records had the title and abstract screened for eligibility. Additional 406 records were excluded for ineligibility based on the initial screen. For the remaining records, full text was sought for retrieval and assessed for inclusion based on the inclusion criteria. References of potentially eligible records were screened for any additional studies not identified by the search strategy which identified 6 additional records. Out of the 148 records sought for full-text retrieval, 136 records were obtained. The remaining 12 records were unobtainable despite contacting the authors and/or journal (Additional file 2: Appendix A2). Finally, 37 records were excluded for reasons shown in Fig. 1, leaving 99 studies fulfilling the inclusion criteria and included in the final analysis.

### Study characteristics

The study characteristics are summarized in Table 1. The included 99 studies were in English language and covered the period from 1981 to 2020. Detailed summary of studies’ characteristics is shown in Additional file 2: Appendix C. Most studies were performed in KSA (n = 75) [10, 21–91], followed by Kuwait (n = 5) [92–96], UAE (n = 5) [97–101], Oman (n = 5) [102–106], Qatar (n = 4) [107–110] and finally Bahrain (n = 3) [111–113]. There was one study that was performed in both Bahrain and KSA [114] and another that included Oman, UAE and Qatar [115]. The average sample size of the country-specific studies varied, and while KSA had the highest number of studies, Bahrain had the largest number of sample size and contributed a higher proportion (45.5%) of studied participants in the GCC. Most studies were cross-sectional in design (n = 63), followed by retrospective cohort (n = 30), case–control (n = 5) and finally one prospective cohort study (n = 1). Most studies were peer-reviewed published studies (n = 94), but 4 studies were published as abstracts and one study was a PhD thesis. Most of the included studies (n = 87) were based entirely or partially in a health-care facility, while five were community-based [32, 33, 55, 67, 71]. ELISA was the most used diagnostic test (71 studies) followed by CMIA (7 studies). All studies reported apparent prevalence only.

**Table 1:** The characteristics and number of hepatitis B virus studies included from some subgroups in the Gulf Cooperation Council countries, number of tested and positive subjects, prevalence (random effect model), and I^2^%

Category	No. of studies	No. tested (%)	No. positive	HBV prevalence % (95% CI)	I^2^%
Overall	99	1,944,200 (100)	30,206	3.05 (2.60, 3.52)	99.6
1. Country (n = 1,934,156)					
Bahrain	4	878,291(45.5)	5068	2.42 (0.23, 6.35)	89.0
KSA	76	656,824 (34.0)	17,933	3.36 (2.86, 3.90)	99.2
Kuwait	5	17,332 (0.8)	308	2.31 (0.47, 5.41)	98.4
Oman	6	8169 (0.4)	248	3.73 (1.85, 6.23)	95.8
Qatar	5	192,799 (10.0)	5771	2.57 (1.62, 3.73)	81.2
UAE	6	180,741 (9.4)	706	0.80 (0.00, 2.99)	99.2
2. Residence status (n = 484,632)					
Nationals	51	303,023 (62.5)	5866	2.85 (2.29, 3.47)	98.7
Expatriates	23	181,609 (37.5)	1219	2.22 (1.26, 3.41)	98.5
3. Age (n = 775,138)					
Adults	82	758,988 (97.9)	15,396	3.05 (2.52, 3.62)	99.4
Children	19	16,150 (2.1)	487	1.93 (0.42, 4.17)	97.3

Category	No. of studies	No. tested	No. positive	HBV prevalence % (95% CI)	I^2^%
4. Gender (n = 251,803)					
Male	42	119,837 (47.6)	3357	2.94 (2.14, 3.85)	98.3
Female	43	131,966 (52.4)	3377	1.73 (1.10, 2.46)	97.9
5. Risk level (n = 1,929,957)					
Average-risk	79	1,909,957	29,222	2.85 (2.39, 3.36)	99.7
High-risk	27	16,110	953	6.12 (3.85, 8.84)	97.3
6. Risk group (n = 770,281)					
Pregnant women	10	17,643	493	2.66 (1.81, 3.67)	92.6
Blood donors	33	731,399	15,713	2.33 (1.67, 3.09)	99.7
Hemodialysis	9	2221	66	5.76 (2.42, 10.18)	87.7
Health-care workers	6	19,018	110	0.86 (0.08, 2.23)	93.3
7. Decade (n = 1,935,906)					
1981–1990	13	42,486	2476	9.38 (7.26, 11.74)	97.5
1990–1999	21	194,457	7602	4.70 (3.76, 5.74)	98.6
2000–2009	34	1,252,240	11,444	2.11 (1.65, 2.63)	99.5
2010–2021	37	446,723	8539	1.56 (1.07, 2.12)	99.3
8. Diagnostic test (n = 1,634,136)					
ELISA	71	1,311,130	18,242	3.24 (2.64, 3.89)	99.5
CMIA	7	323,006	7059	1.06 (0.04, 2.09)	99.7

Cochran’s *p*-values were < 0.001 for all the subgroups

### Overall prevalence and by country

**Fig. 2:**
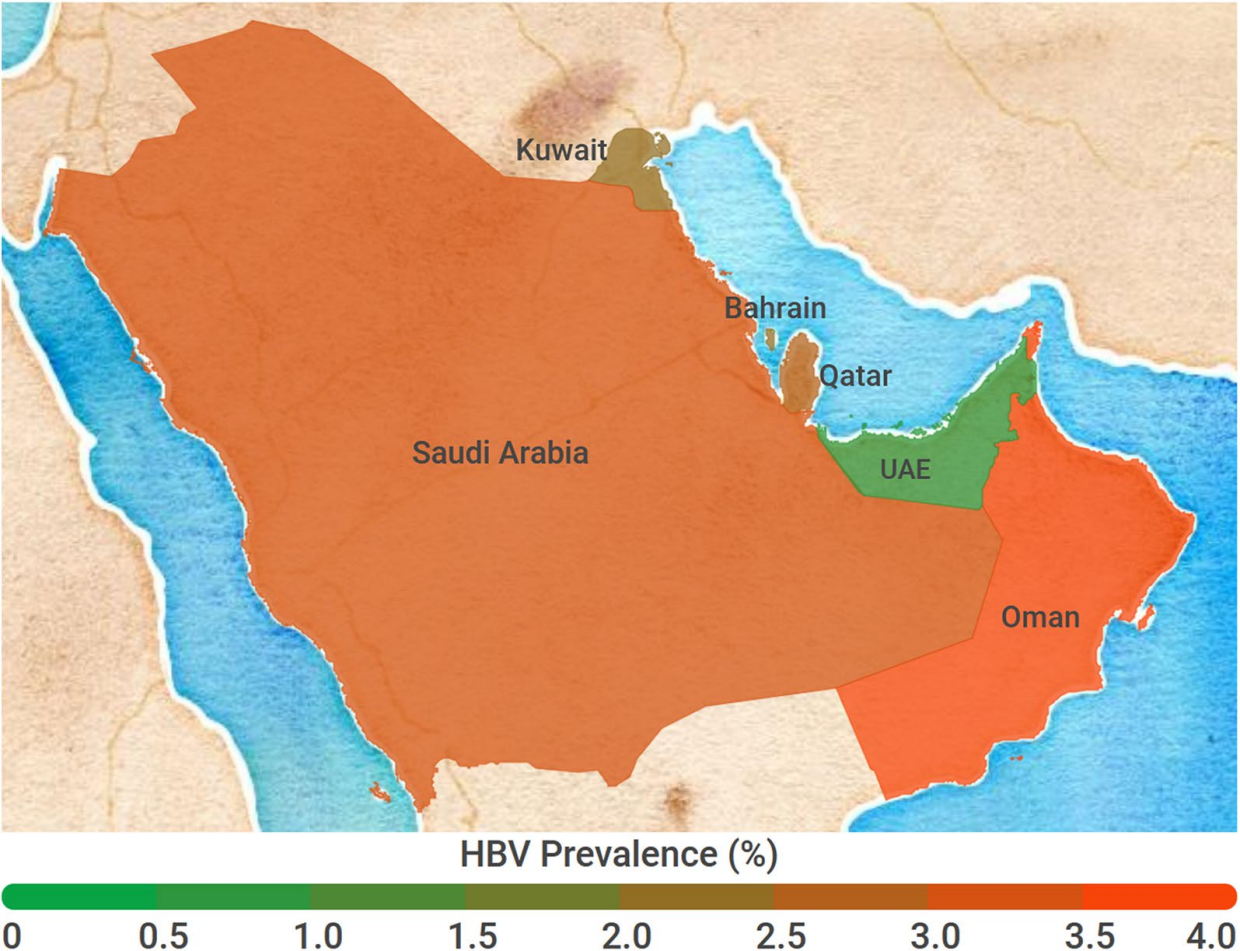
Geographical variation in hepatitis B virus prevalence among member countries of the Gulf Cooperation Council (source of figure: infogram.com)

The estimated overall HBV prevalence in the GCC region was 3.05% (95% CI 2.60, 3.52%). The prevalence rates varied according to countries (Table 1, Forest plots and Additional file 2: Appendix D). The prevalence of HBV infection among the GCC countries varied between 0.80% in UAE to 3.73% in Oman (Fig. 2).

### True prevalence

Calculating the true HBV prevalence resulted in lower estimate in the GCC and all individual countries (Table 2).

**Table 2:** Apparent and true hepatitis B virus prevalence estimates in the Gulf Cooperation Council region and member-countries

GCC country	Apparent prevalence (95% CI)	True prevalence (95% CI)
All	3.05 (2.60, 3.52)	1.67 (1.66, 1.68)
Bahrain	2.42 (0.23, 6.35)	0.93 (0.66, 1.22)
KSA	3.36 (2.86, 3.90)	2.02 (1.70, 2.34)
Kuwait	2.31 (0.47, 5.41)	0.81 (0.38, 1.24)
Oman	3.73 (1.85, 6.23)	2.44 (2.38, 2.50)
Qatar	2.57 (1.62, 3.73)	1.11 (0.93, 1.29)
UAE	0.80 (0.00, 2.99)	0.00 (0.00, 0.19)

### Prevalence over time

**Fig. 3:**
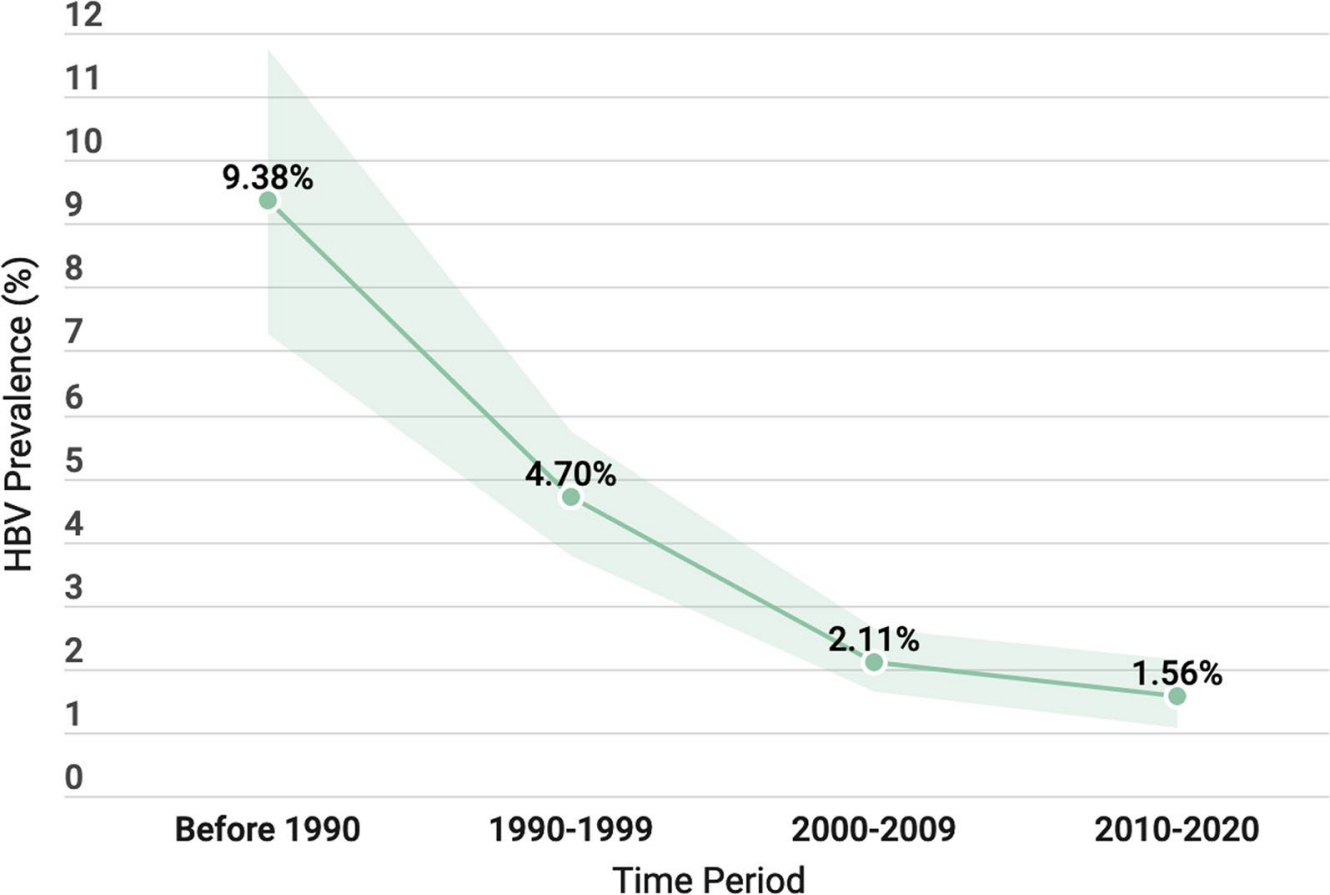
The trend of hepatitis B virus infection prevalence over time in the Gulf Cooperation Council region (the shaded area represents the 95%CI)

Prior to the introduction of HBV vaccine in the early 1990s, the GCC region had a HBV prevalence of 9.38%, placing it in the high-endemicity category. The region moved to the intermediate-endemicity category during the 1990s (HBV prevalence 4.70%). After the Millennium, the GCC region moved to the low-intermediate endemicity with HBV prevalence of 2.11%. Finally, the region moved to the low-endemicity category after 2010 with the latest HBV infection estimate of 1.56% (Fig. 3).

### Prevalence by risk group and setting

**Fig. 4:**
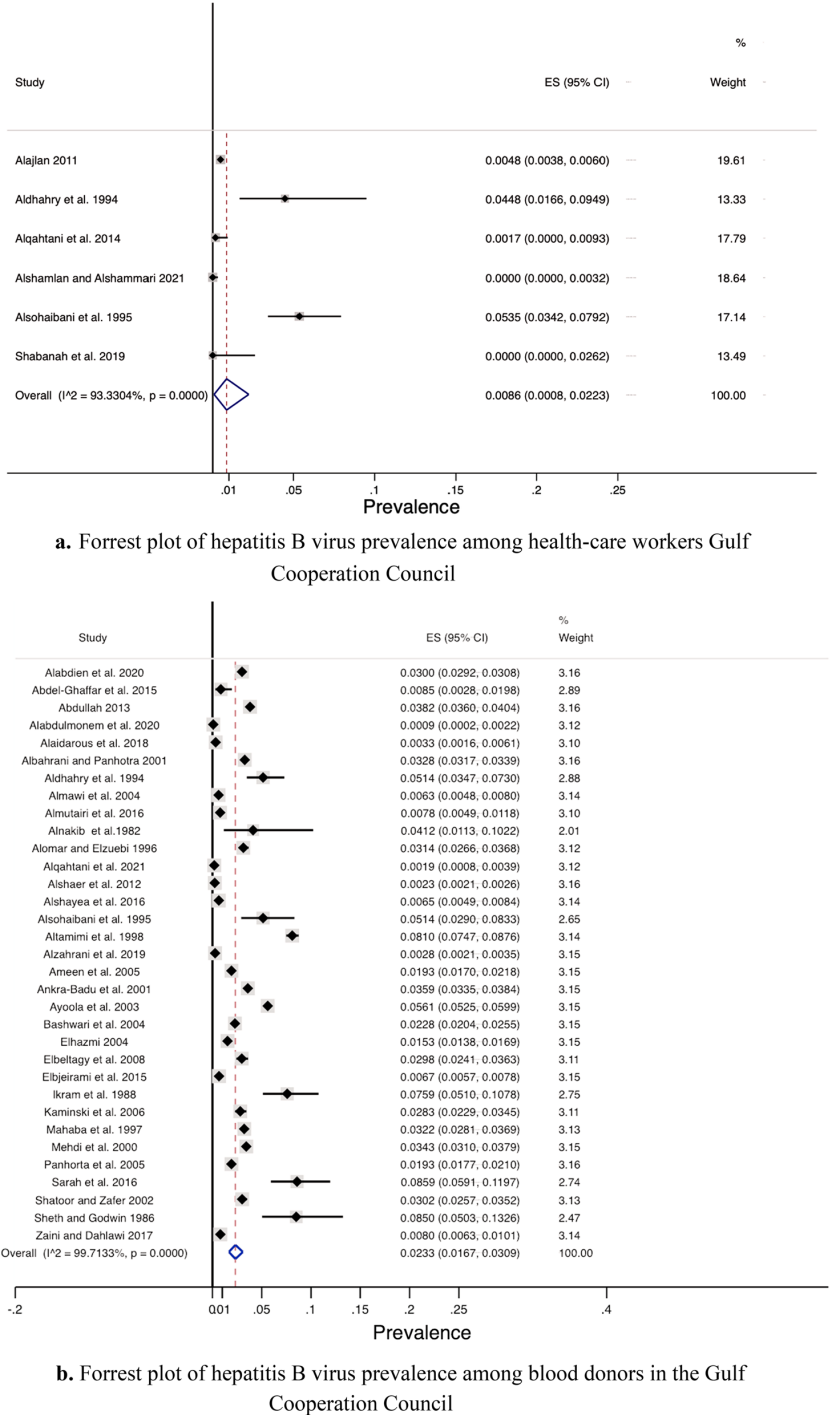

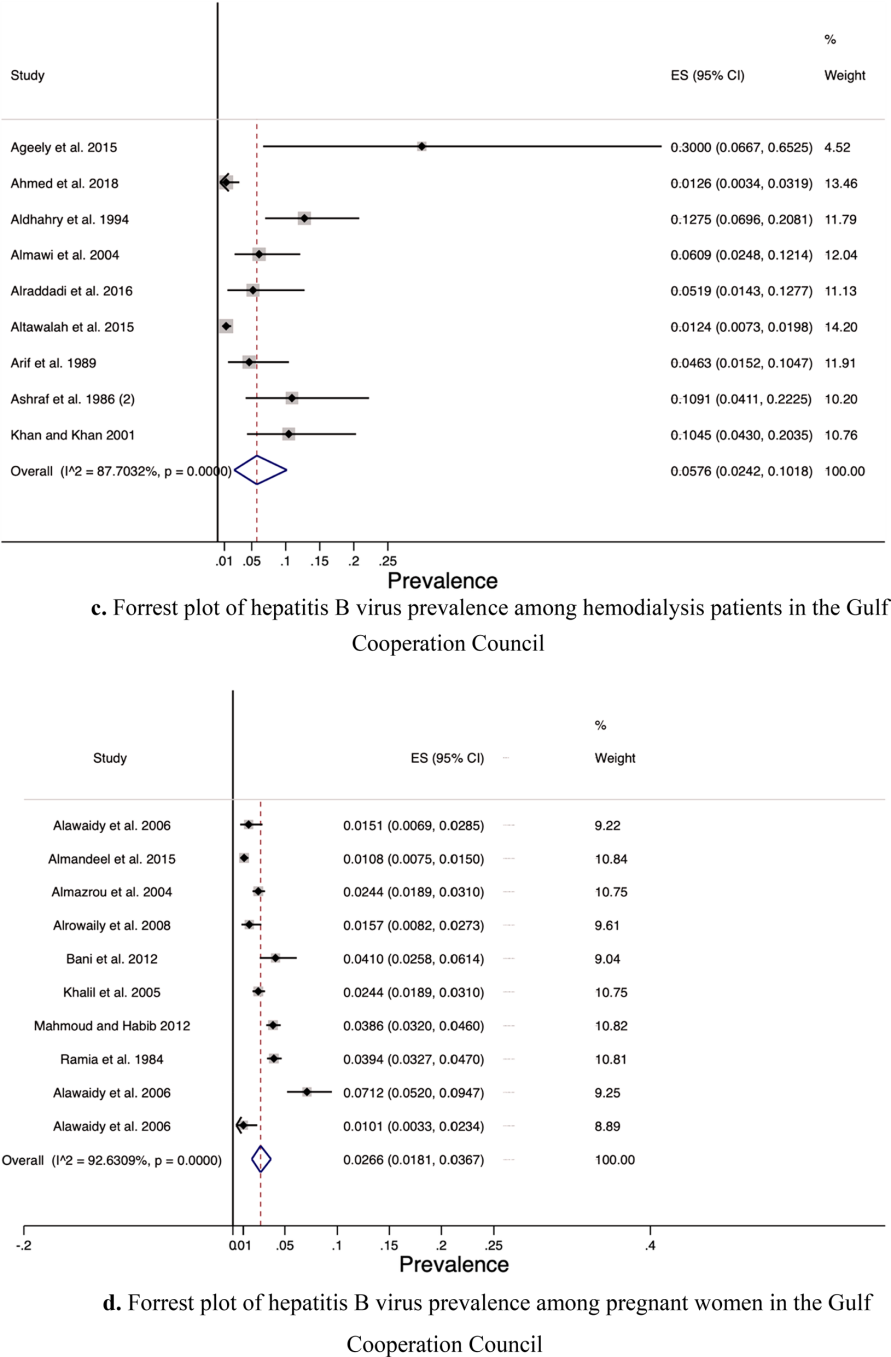
**a** Forrest plot of hepatitis B virus prevalence among health-care workers Gulf Cooperation Council. **b** Forrest plot of hepatitis B virus prevalence among blood donors in the Gulf Cooperation Council. **c** Forrest plot of hepatitis B virus prevalence among hemodialysis patients in the Gulf Cooperation Council. **d** Forrest plot of hepatitis B virus prevalence among pregnant women in the Gulf Cooperation Council

Among the risk groups, the prevalence was lowest among health-care workers (0.86%), followed by blood-donors (2.33%), pregnant women (2.66%), and finally hemodialysis patients (5.76%) (see Fig. 4a–d). The pooled HBV prevalence from the community-based studies was 4.21% (95% CI 1.39, 8.46).

#### Quality assessment and sensitivity analysis

The overall quality of the individual studies and detailed description are shown in Additional file 2: appendices C and E. Almost half of the included studies were of poor quality (n = 49, 49.5%). Most studies used convenient sampling (n = 94), did not address missing data (n = 93), report confidence intervals (n = 95), potential source of bias (n = 82) or estimate the random variability in the data (n = 87).

Since the meta-analysis of the overall population exhibited a considerable heterogeneity, subgroup analysis was done (Table 1). The heterogeneity remained high among all tested subgroups with I^2^ ranging between 81.2% and 99.7%. When low-quality studies were excluded in the sensitivity analysis, the HBV prevalence was found to be 2.96% (95% CI 2.40, 3.58) using random-effect model (I^2^ = 99.6%). Hence, the quality of the studies had little effect on the overall estimated HBV prevalence.

### Publication bias

**Fig. 5:**
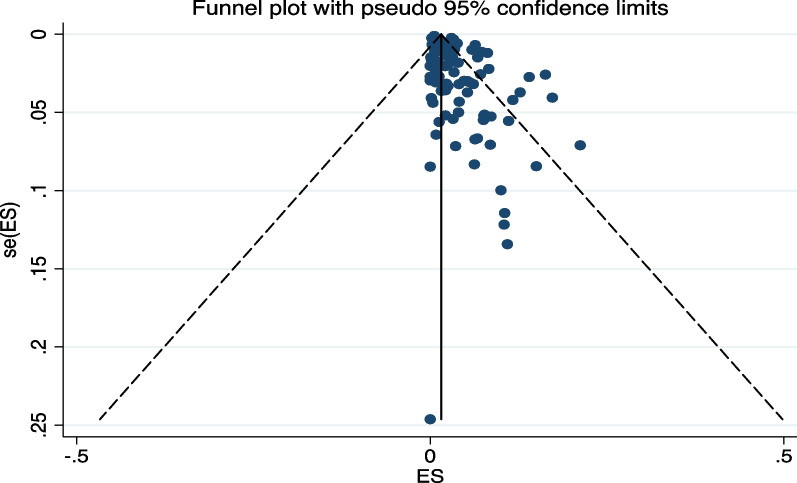
Funnel plot of included studies showing asymmetry in the distribution of the dots consistent with publication bias

Asymmetrical distribution and gaps in the funnel plot indicate the presence of publication bias (Fig. 5), confirmed more objectively with the Egger’s test p-value < 0.001.

## Discussion

Interest in estimating the prevalence of HBV has been increasing over the last four decades in the GCC. This is shown by the five-fold increase in the number of publications addressing the prevalence of this infection in the region from only 11 studies covering the period of 1980–1990 to 50 studies published in the period 2010–2021. However, there is a clear discrepancy among the individual member countries of the GCC when it comes to the number of papers published in this field. KSA showed early and increasing interest in estimating the burden of HBV infection and this is manifested by the large number of publications (n = 76) spanning the period from 1982 to 2020 and matching the population trend in the GCC (71% of the total population). On the other hand, the other member countries had lower number of publications in this field (4–6 studies per country) and the least populated countries (Bahrain and Qatar) employed larger samples in their studies. However, it is encouraging to observe that these studies were published more recently confirming the recognition of the importance of HBV infection as a global and regional health problem.

In the current study, the overall HBV apparent and true prevalence, as measured by HBsAg, was 3.05% and 1.67% respectively in the GCC region using the data available from the 99 included studies published over the last four decades. However, when only the most recent studies (2010 to 2020) were analyzed separately, the overall HBV apparent prevalence was found to be 1.56%. This figure is much lower than the estimated WHO prevalence and the narrow confidence intervals for the prevalence (95% CI 1.07, 2.12) provides more evidence that the current HBV prevalence estimate is a better one and supports the conclusion that the GCC region is a low-endemicity region for HBV infection. Furthermore, the study showed variations in the prevalence of HBV among the different countries of the GCC. The prevalence was lowest in the UAE (0.80%) while it was highest in Oman (3.73%). The UAE stringent visa medical requirements regarding HBV that include retesting of certain work permit holders may be responsible [116]. However, The overlapping 95% confidence intervals of the prevalence between the GCC countries is suggestive of resemblance.

Even though the expatriates form 49% of the GCC population [117], this subgroup was underrepresented in the included studies (23 studies, 37.5% of total participants). Similarly, children were included in only 5 studies accounting for 2.1% of total participants in the current study. While suspected reluctancy of some expatriates to participate fearing negative implications to their work permit visa may be blamed, difficulty in recruiting children into studies, and/or underestimation of the importance of HBV infection among this group may be the reason. On the contrary, tested female proportion (45.5%) was overrepresented compared to both male and children GCC proportions. A clearer example of using convenient sampling is utilizing blood bank records to estimate HBV prevalence. This method will selectively favour healthy volunteers since older and unhealthy individuals are less likely to donate blood. Furthermore, in the GCC region, males are the predominant blood donors, resulting in overrepresentation of this gender in studies employed blood donors compared to females. All these factors and deficiencies were evident in 50% of the included studies and may resulted in methodologically weak level of evidence.

Children had lower prevalence of HBV (1.93%) compared to adults (3.05%) but the confidence intervals of both overlapped. Although, children constitute 22.1% of the population in the GCC, only 2.1% of the included studies participants (19 studies) were children [118]. Hence, this might be an imprecise estimate of the prevalence of HBV in children, and further larger pediatric studies are required to address this issue. A lower prevalence of HBV is expected as children are less likely to belong to high-risk population (e.g. PWID) or exposed to activities that increase the risk of HBV infection, such as needle stick injuries, compared to adults. However, this difference could be another reflection of the success of early interventions to reduce the risk of HBV infection. The current hepatology guidelines [1] suggest the initiation of anti-viral therapy for mothers with HBV infection during pregnancy and early administration of HBV vaccine (within 24 h of birth) to reduce the risk HBV infection. This practice may have resulted in lower rate of vertical transmission, hence lowering the risk of infection among children.

The results of this review demonstrated a decreasing prevalence over time (Fig. 3) providing evidence of the efficacy of the national strategies including the HBV vaccination programs aiming to mitigate the spread of the virus in the GCC communities and reducing the burden of this infectious disease. In fact, prior to the introduction of the vaccine into the GCC region, HBV had overall prevalence of 9.38%, making it a high-prevalence region. But after incorporating the HBV vaccine into the vaccination programs after 1990, the prevalence have steadily decreased over the following three decades supporting the WHO recommendation for universal HBV vaccination [2]. The most recent estimate is 1.56% placing the GCC in the “low-endemicity” category. This transition will ultimately lead to decrease morbidity and mortality related to HBV, with subsequent reduction in health-care cost. Furthermore, the reduction of HBV prevalence in this region suggests that the GCC countries are in the correct pathway to eradicate HBV by 2030 in keeping with the GHSS target [2].

## Strengths, limitations, and risk of biases

This study has several strengths. First, the search strategy included three commonly used databases along with searching references of reviewed studies. The exclusion criteria were kept to a minimum to increase sensitivity of the search and not to miss eligible articles. This ultimately led to the inclusion of 99 studies translating into over 1.9 million participants. This large number of included participants lead to improved HBV prevalence estimation, decreased the possibility of chance finding and narrowed the confidence intervals obtained for the main outcome. In addition, this thorough search led to the inclusion of studies that covered all member countries of the GCC region, different populations and time frames, increasing the generalizability of the findings. Another strength of the study was the use of quality assessment tool for the assessment of the quality of included studies and risk of bias. This assessment allowed the recognition of methodologically weak studies and performing sensitivity analysis excluding these studies, further confirming the observed prevalence. The use of a validated and reliable blood test to detect HBsAg and ascertain the outcome (HBV infection) as part of the inclusion criteria helped to reduce the risk of information bias. This is important since using non-accurate methods for detection of HBV infection (e.g. physical examination only or non-validated tests) may have resulted in biased results. Also, we used the random-effect model to account for heterogeneity. This is the first study to consider the unique demographic characteristics of the GCC countries population and the work permit health requirement in interpreting the results and calculate true prevalence.

However, this study has some limitations. The major limitation is related to the quality of the included studies. The overall quality assessment showed that half of the included studies were of poor quality, mainly related to the sampling technique used, adjustment for confounders and accounting for missing participants. Only 5 studies [32, 33, 55, 67, 71] utilized community-sampling which normally would provide better estimate of the general population prevalence of HBV. The included studies had evidence of significant heterogeneity as measured by I^2^ test and Cochran’s Q test. The source of the heterogeneity could be related to using different population groups (e.g. blood donors, hemodialysis, pregnant women). This issue of high heterogeneity is well-described for descriptive studies since they rely mostly on retrospective observational studies where controlling for different sources of bias might be more difficult compared to a controlled interventional study [119]. In addition, another source of heterogeneity is the inclusion of different time frames to estimate the pooled HBV prevalence. Methodologically, only one author screened and selected the available literature applying inclusion and exclusion criteria. While this is not in line with the best practices required for a systematic review, it was because of the nature and the specific requirements of an individual work within an academic path, and it was mitigated by an independent supervision by the second author in view of the peer review process.

The funnel plot test result and the low *p*-value of the Egger’s test could represent a true evidence of publication bias where studies that reported higher prevalence of HBV infection were more likely to be published. In addition, there were 12 potentially eligible studies that were unretrievable (Additional file 2: Appendix A2). In theory this could have introduced selection bias. However, this is less likely to have a significant impact on the overall estimate of this review given the large number of included studies (n = 99). Furthermore, most of the missing articles were published in the pre-2000 period, having even less impact on the estimate of the current HBV prevalence.

## Conclusions and recommendations

The overall apparent prevalence of HBV infection in the GCC is 3.05% (95% CI 2.60%, 3.52) and the true prevalence is 1.67% (95% CI 1.66%, 1.68). Over the last four decades the overall apparent prevalence of HBV infection in the GCC region decreased from 9.38% (95% CI 7.26, 11.74) before 1990 to 1.56% (95% CI 1.07, 2.12) during the period 2010 to 2020. Therefore, the GCC region has successfully moved from high-endemicity region to low-endemicity region. However, due to poor methodology of the included studies, further high-quality, community-based studies are needed with focus on the prevalence in the general population.

## Supplementary Information

**Additional file 1.** PRISMA checklist.

**Additional file 2**. Appendix.

## Data Availability

All data and statistical codes used to generate the results will be available on request to the corresponding author.
