# Supplementary material for: Prevalence of Hepatitis B Virus infection in the Gulf Cooperation Council: a systematic review and meta-analysis
Source: BMC Infect Dis. 2022 Nov 7;22:819. doi: 10.1186/s12879-022-07806-4 (PMC9641946; doi:10.1186/s12879-022-07806-4)
Supplement: Supplementary file 2 — Additional file 2. Appendix. [file 12879_2022_7806_MOESM2_ESM.docx]

**Appendix A1. Details of search strategy by Database**

**EMBASE**

| **#** | **Query** | **Results from 30 Jul 2021** |
| --- | --- | --- |
| **1** | **((HBV or hepatitis B or hepatitis B virus or hepatitis B surface antigen or HBsAg) adj3 infect*)** | **47,948** |
| **2** | **exp hepatitis B/** | **110,023** |
| **3** | **(GCC or gulf council cooperation or Arabian gulf or Kuwait* or Saudi* or KSA or kingdom of Saudi or United Arab Emirates or UAE or Emirati or Bahrain* or oman* or Qatar*)** | **61,737** |
| **4** | **exp Kuwait/** | **5,448** |
| **5** | **exp Saudi Arabia/** | **24,471** |
| **6** | **exp United Arab Emirates/** | **4,364** |
| **7** | **exp Oman/** | **3,284** |
| **8** | **exp Bahrain/** | **1,605** |
| **9** | **exp Qatar/** | **3,433** |
| **10** | **(prevalence or epidemiology or seroprevalence or Seroepidemiology)** | **2,349,912** |
| **11** | **exp prevalence/** | **834,787** |
| **12** | **exp epidemiology/** | **3,881,699** |
| **13** | **exp seroepidemiology/** | **4,583** |
| **14** | **1 or 2** | **123,813** |
| **15** | **3 or 4 or 5 or 6 or 7 or 8 or 9** | **62,100** |
| **16** | **10 or 11 or 12 or 13** | **4,708,497** |

| **17** | **14 and 15 and 16** | **336** |
| --- | --- | --- |

**Medline**

| **#** | **Query** | **Results from 30 Jul 2021** |
| --- | --- | --- |
| **1** | **((HBV or hepatitis B or hepatitis B virus or hepatitis B surface antigen or HBsAg) adj3 infect*)** | **32,376** |
| **2** | **exp Hepatitis B, Chronic/ or exp Hepatitis B/ or exp Hepatitis B virus/** | **69,111** |
| **3** | **(GCC or gulf council cooperation or Arabian gulf or Kuwait* or Saudi* or KSA or kingdom of Saudi or United Arab Emirates or UAE or Emirati or Bahrain* or oman* or Qatar*)** | **46,469** |
| **4** | **exp Kuwait/** | **3,290** |
| **5** | **exp Saudi Arabia/** | **14,654** |
| **6** | **exp United Arab Emirates/** | **2,286** |
| **7** | **exp Oman/** | **1,669** |
| **8** | **exp Bahrain/** | **647** |
| **9** | **exp Qatar/** | **1,410** |
| **10** | **(prevalence or epidemiology or seroprevalence or Seroepidemiology)** | **2,282,547** |
| **11** | **exp Prevalence/** | **313,135** |
| **12** | **exp Epidemiology/** | **27,566** |
| **13** | **exp Seroepidemiologic Studies/** | **25,153** |
| **14** | **1 or 2** | **77,566** |
| **15** | **3 or 4 or 5 or 6 or 7 or 8 or 9** | **46,469** |
| **16** | **10 or 11 or 12 or 13** | **2,284,197** |

| **17** | **14 and 15 and 16** | **206** |
| --- | --- | --- |

**Global Health**

| **#** | **Query** | **Results from 30 Jul 2021** |
| --- | --- | --- |
| **1** | **((HBV or hepatitis B or hepatitis B virus or hepatitis B surface antigen or HBsAg) adj3 infect*)** | **16,970** |
| **2** | **exp Hepatitis B/** | **35,878** |
| **3** | **(GCC or gulf council cooperation or Arabian gulf or Kuwait* or Saudi* or KSA or kingdom of Saudi or United Arab Emirates or UAE or Emirati or Bahrain* or oman* or Qatar*)** | **19,488** |
| **4** | **exp Kuwait/** | **2,167** |
| **5** | **exp Saudi Arabia/** | **11,683** |
| **6** | **exp United Arab Emirates/** | **1,677** |
| **7** | **exp Oman/** | **1,542** |
| **8** | **exp Bahrain/** | **648** |
| **9** | **exp Qatar/** | **994** |
| **10** | **(prevalence or epidemiology or seroprevalence or Seroepidemiology)** | **601,384** |
| **11** | **exp Epidemiology/** | **367,891** |

| **12** | **1 or 2** | **37,448** |
| --- | --- | --- |
| **13** | **3 or 4 or 5 or 6 or 7 or 8 or 9** | **19,488** |
| **14** | **10 or 11** | **601,384** |
| **15** | **12 and 13 and 14** | **167** |

**Appendix A2. Missing full-text articles**

Articles that full-text was missing and not able to retrieve despite contacting authors and/or journal:

1. Eifan SA, Mohamed HAK. Seroprevalence of hepatitis B and C virus in premarital screening program in and around albaha region of Saudi Arabia. Journal of Pure and Applied Microbiology 2014;8:1591-1595.
2. el-Hazmi MA, Ramia S. Frequencies of hepatitis B, delta and human immune deficiency virus markers in multitransfused Saudi patients with thalassaemia and sickle-cell disease. *J Trop Med Hyg*. 1989;92(1):1-5.
3. el-Hazmi MA. Hepatitis B virus in Saudi Arabia. *J Trop Med Hyg*. 1989;92(1):56-61.
4. Ramia S, Hossain A, Bakir TM, Waller DK, Vivian PA. Prevalence and subtype of hepatitis B surface antigen (HBsAg) in the Saudi population. *Trop Geogr Med*. 1986;38(1):63-69.
5. Safi MA, Ghaznawi HT, Arafa MS, Moselhi M. An immunoserological study among blood donors of Makkah region, Saudi Arabia. *J Egypt Soc Parasitol*. 1989;19(1):91-99.
6. Al-Nakib W, Bashir AA. Prevalence of hepatitis B (surface) antigen (HBsAg) among healthy blood donors in Kuwait: evaluation of a passive-haemagglutination (PHA) test. Journal of the Kuwait Medical Association 1977;11:137-144.
7. Arif M. Age specific markers to hepatitis B and hepatitis D viruses in the Saudi population: prevalence and epidemiological significance. Emirates Medical Jounral 1991
8. Bate CM. Hepatitis B in the Emirates. Emirates Medical Jounral 1980
9. Fathalla SE, Namnyak SS, Al Jama A, Rabaria Bautista MM. The prevalence of hepatitis B surface antigen in healthy subjects residing in the Eastern Province of Saudi Arabia. *Saudi Med J.* 1985; 6: 236-239
10. Fathalla SE, Al-Jama AA, Al-Sheikh IH, et al. Seroepidemiological prevalence of hepatitis B virus markers in Eastern Saudi Arabia. *Saudi Med J*. 1998;19(3):283-288.
11. Fikri M, Dajani AI and Fouad WS. Epidemiology of HBV serological markers in public health workers in the Sharjah medical area. Emirates Medical Jounral 1987
12. Kashem A and Karim MR. Prevalence of Hepatitis B and C among Hemodialysis Patients in Najran of Saudi Arabia. Bangladesh Renal Journal 2002

**Appendix B: Quality Assessment Tools**

| **For Case-Control studies** | | | | |
| --- | --- | --- | --- | --- |
| 1 | Cases and controls are taken from comparable populations | Yes | No | NA |
| 2 | The same exclusion criteria are used for both cases and controls |  |  |  |
| 3 | Cases are clearly defined and differentiated from controls |  |  |  |
| 4 | It is clearly established that controls are non-cases |  |  |  |
| 5 | Measures will have been taken to prevent knowledge of primary exposure influencing case ascertainment |  |  |  |
| 6 | Exposure status is measured in a standard, valid and reliable way |  |  |  |
| 7 | The main potential confounders are identified and taken into account in the design and analysis |  |  |  |
| 8 | Potential confounders are defined and taken into account |  |  |  |
| 9 | Statistical methods are described clearly |  |  |  |
| 10 | Statistical methods are appropriate |  |  |  |
| 11 | Missing data were addressed accurately |  |  |  |
| 12 | Confidence intervals are provided in statistical analysis |  |  |  |
| 13 | Sources of bias are mentioned clearly |  |  |  |
| 14 | The study provides estimates of the random variability in the data for the main outcomes |  |  |  |

**Appendix B continued**

| **For cohort studies** | | | | |
| --- | --- | --- | --- | --- |
| NO | Item | Yes | No | NA |
| 1 | The two groups being studied are selected from source populations that are comparable in all respects other than the factor under investigation. |  |  |  |
| 3 | The likelihood that some eligible subjects might have the outcome at the time of enrolment is assessed and taken into account in the analysis. |  |  |  |
| 5 | Comparison is made between full participants and those lost to follow up, by exposure status |  |  |  |
| 7 | The assessment of outcome is made blind to exposure status. |  |  |  |
| 8 | Where blinding was not possible, there is some recognition that knowledge of exposure status could have influenced the assessment of outcome. |  |  |  |
| 9 | The measure of assessment of exposure is reliable. |  |  |  |
| 10 | Evidence from other sources is used to demonstrate that the method of outcome assessment is valid and reliable. |  |  |  |
| 11 | Exposure level or prognostic factor is assessed more than once. |  |  |  |
| 12 | The main potential confounders are identified and taken into account in the design and analysis. |  |  |  |
| 13 | Statistical methods are described clearly |  |  |  |
| 14 | Statistical methods are appropriate |  |  |  |
| 15 | Missing data were addressed accurately |  |  |  |
| 16 | Confidence intervals are provided in statistical analysis |  |  |  |
| 17 | Sources of bias are mentioned clearly |  |  |  |
| 18 | The study provides estimates of the random variability in the data for the main outcomes |  |  |  |

# 2

**Appendix B continued**

|  | **Checklist for the assessment of the quality of cross-sectional study** |  |  |  |
| --- | --- | --- | --- | --- |
| **No** | **Item** | **Yes** | **No** | **NA** |
| 1 | Sample is representative to the entire population from which it was recruited |  |  |  |
| 2 | Exposure status is measured in a standard, valid and reliable way |  |  |  |
| 3 | The main potential confounders are identified and taken into account in the design and analysis |  |  |  |
| 4 | Potential confounders are defined and taken into account |  |  |  |
| 5 | Statistical methods are described clearly |  |  |  |
| 6 | Statistical methods are appropriate |  |  |  |
| 7 | Missing data were addressed accurately |  |  |  |
| 8 | Confidence intervals are provided in statistical analysis |  |  |  |
| 9 | Sources of bias are mentioned clearly |  |  |  |
| 10 | The study provides estimates of the random variability in the data for the main outcomes |  |  |  |

# 3

**Appendix C. Detailed description of the baseline characteristics of the included studies grouped by country (n=99)**

| **Country** | **Author/year** | **Study type** | **Region** | **Enrollment**  **period** | **Population**  **characteristics** | **Pop.**  **Risk** | **Study**  **setting** | **No.**  **included** | **Adults**  **(%)** | **Male**  **(%)** | **National**  **(%)** | **Test**  **used** | **Quality** |
| --- | --- | --- | --- | --- | --- | --- | --- | --- | --- | --- | --- | --- | --- |
| **KSA** | Abdel-Ghaffar  et al. 2015 | Cross-  sectional | Al-  Quwayiyah | 2014 | Blood donors  Patients | Low | Health  facility | 2215 | 2215  (100%) | 1475  (66.6%) | 982  (44.3%) | ELISA | High |
|  | Abdullah 2013 | Cross-  sectional | Jazan | 2004-2009 | Blood donors | Low | Health  facility | 29949 | 29949  (100%) | NR | NR | ELISA | Low |
|  | Abdullah 2018 | Cross-  sectional | Jazan | 2014-2015 | Pre-marital | Low | Health  facility | 7826 | 7826  (100%) | 3913  (50%) | 7826  (100%) | ELISA | Low |
|  | Ageely 2015 | Cross-  sectional | Jazan | 2009 | General  population | Low | Health  facility | 2041 | 1808  (88.6%) | 912  (44.7%) | NR | Rapid  Test | High |
|  | Ahmed et al.  2018 | Cross-  sectional | Abha | 2016 | Hemodialysis | High | Health  facility | 318 | 318  (100%) | 200  (62.9%) | NR | ELISA | Low |
|  | Alabdulmonem  et al. 2020 | Retrospective  cohort | Buraidah | 2017-2018 | Blood donors | Low | Health  facility | 4590 | 4590  (100%) | 3809  (83.0%) | NR | Rapid  test | Low |
|  | Aladmawy et  al. 1987 | Cross-  sectional | Alkharj | 1987 | General  population | Low | Health  facility | 7894 | NR | NR | NR | RPHA | Low |
|  | Alaidarous et  al. 2018 | Cross-  sectional | Majmaah | 2015-2017 | Blood donors | Low | Health  facility | 3028 | 3028  (100%) | NR | NR | ELISA | Low |
|  | Alajlan 2011 | Cross-  sectional | National | 2000-2007 | Medical  students | Medium | College | 16570 | 16570  (100%) | 9852  (59.5%) | NR | ELISA | High |
|  | Albadran et al.  2017 | Retrospective  cohort | Riyadh | 2002-2005  2012-2015 | In-Vitro  fertilization | Low | Health  facility | 10189 | 10189  (100%) | NR | 10189  (100%) | NR | Low |
|  | Albahrani and  Panhotra 2001 | Retrospective  cohort | Alhasa | 1987-1999 | Blood donors | Low | Health  facility | 95539 | 95539  (100%) | NR | NR | ELISA | Low |
|  | Alfaleh et al.  1992 | Cross-  sectional | National | 1989-1990 | General  population | Low | Community | 4575 | 0 (0%) | 2458  (53.7%) | 4575  (100%) | ELISA | High |
|  | Alfaleh et al.  1999 | Cross-  sectional | National | 1997 | General  population | Low | Community | 4791 | 0 (0%) | NR | 4791  (100%) | ELISA | Low |
|  | Alfaleh et al.  2008 | Cross-  sectional | National | 2007-2008 | School children | Low | School | 1355 | 0 (0%) | 689  (50.8%) | 1355  (100%) | ELISA | High |
|  | Alfawaz and Ramia 1993 | Cross- sectional | Riyadh | 1993 | Healthy  Hemolytic anemia | Low High | Health facility | 195 | 0 (0%) | NR | NR | ELISA | Low |
|  | Alfreihi 1993 | Case-control | Dammam | 1993 | Schistosomiasis | High | Health  facility | 140 | 140  (100%) | NR | 32  (22.9%) | ELISA | High |
|  | Alghamdi and  Safi 2015 | Cross-  sectional | Makkah | 2015 | Pilgrims | Low | Health  facility | 982 | 982  (100%) | 798  (81.3%) | 15  (1.5%) | ELISA | High |
|  | Alghonaim et al. 2012 | Cross- sectional | Al- Quwayiyah | 2012 | General population Blood donors  Pre-marital | Low | Health facility | 2400 | 2400  (100%) | NR | NR | ELISA | Low |

|  | Alhumayed  2016 | Cross-  sectional | Aseer | 2014 | General  population | Low | Health  facility | 1071 | 938  (87.6%) | NR | NR | ELISA | High |
| --- | --- | --- | --- | --- | --- | --- | --- | --- | --- | --- | --- | --- | --- |
|  | Alhymayed et  al. 2017 | Cross-  sectional | Aseer | 2013 | General  population | Low | Health  facility | 10234 | 9841  (96.2%) | 4842  (47.3%) | NR | ELISA | High |
|  | Alhymayed  2017 | Cross-  sectional | Aseer | 2014 | General  population | Low | Health  facility | 1532 | NR | 876  (57.2%) | NR | ELISA | High |
|  | Alhuraiji et al.  2014 | Retrospective  cohort | Riyadh | 1985-2010 | HIV | High | Health  facility | 341 | 341  (100%) | NR | NR | NR | High |
|  | Aljarbou 2012 | Cross-  sectional | Qassim | 2008-2010 | Pre-marital | Low | Health  facility | 8082 | 8082  (100%) | 4041  (50%) | NR | ELISA | Low |
|  | Almandeel et  al. 2015 | Cross-  sectional | Riyadh | 2010-2011 | Pregnant | Low | Health  facility | 3242 | 3242  (100%) | 0 (0%) | 3242  (100%) | ELISA | Low |
|  | Almazrou et al.  2004 | Cross-  sectional | National | 2003 | Pregnant | Low | Health  facility | 2664 | 2664  (100%) | 0 (0%) | 2664  (100%) | ELISA | High |
|  | Almutairi et al.  2016 | Cross-  sectional | Albaha | 2009-2011 | Blood donors | Low | Health  facility | 2807 | 2807  (100%) | 2807  (100%) | NR | ELISA | High |
|  | Alomar and  Elzuebi 1996 | Cross-  sectional | Albaha | 1993-1994 | Blood donors | Low | Health  facility | 4683 | 4683  (100%) | NR | 3419  (73.0%) | ELISA | Low |
|  | Alqahtani et al.  2014 | Cross-  sectional | Najran | 2014 | Health-care  workers | Medium | Health  facility | 600 | 600  (100%) | 280  (46.7%) | 345  (57.5%) | ELISA | High |
|  | Alqahtani et al.  2021 | Cross-  sectional | Majmaah | 2019-2020 | Blood donors | Low | Heath  facility | 3733 | 3733  (100%) | 3645  (97.6%) | NR | ELISA | High |
|  | Alraddadi et al.  2016 | Cross-  sectional | Jeddah | 2012-2013 | General  population | Low | Health  facility | 5584 | 5584  (100%) | 1679  (30.1%) | 5584  (100%) | ELISA | High |
|  | Alrowaily et al.  2008 | Cross-  sectional | Riyadh | 2005-2006 | Pregnant | Low | Health  facility | 762 | 762  (100%) | 0 (0%) | NR | ELISA | High |
|  | Alshamlan and Alshammari  2021 | Cross- sectional | Dammam | 2000 | Medical students | Medium | College | 1145 | 1145  (100%) | 516  (45.1%) | NR | NR | High |
|  | Alshayea et al.  2016 | Cross-  sectional | Riyadh | 2011-2012 | Blood donors | Low | Health  facility | 8601 | 8601  (100%) | NR | NR | CMIA | Low |
|  | Alsohaibani et al. 1995 | Cross- sectional | Riyadh  Al-Khobar | 1992-1994 | Blood donors Medical students  Medical staff | Low Medium | Health facility College | 722 | 722  (100%) | 515  (71.3%) | 722  (100%) | ELISA | High |
|  | Alshomrani  2015 | Retrospective  cohort | Riyadh | 2006-2012 | Heroin addicts | High | Health  facility | 378 | 378  (100%) | 378  (100%) | NR | ELISA | High |
|  | Alswaidi and  O’Brien 2009 | Cross-  sectional | National | 2008 | Pre-marital | Low | Community | 74662 | 74662  (100%) | NR | 74662  (100%) | CMIA | High |
|  | Altamimi et al.  1998 | Cross-  sectional | Riyadh | 1992-1995 | Blood donors | Low | Health  facility | 7013 | 7013  (100%) | NR | NR | ELISA | Low |
|  | Althaqafy et al.  2013 | Cross-  sectional | Jeddah | 2009 | Military | Low | Military | 400 | 400  (100%) | 400  (100%) | 400  (100%) | ELISA | High |
|  | Alwayli 2009 | Retrospective  cohort | Riyadh | 1994-2007 | General  population | Low | Health  facility | 79858 | NR | 20288  (25.4%) | NR | NR | Low |

|  | Alzahrani et al.  2019 | Retrospective  cohort | Dammam | 2011-2015 | Blood donors | Low | Health  facility | 22842 | 22842  (100%) | NR | 22842  (100%) | CMIA | Low |
| --- | --- | --- | --- | --- | --- | --- | --- | --- | --- | --- | --- | --- | --- |
|  | Ankra-Badu et  al. 2001 | Retrospective  cohort | Al-Khobar | 1992-1996 | Blood donors | Low | Health  facility | 23043 | 23043  (100%) | NR | 18195  (80.0%) | ELISA | Low |
|  | Arif et al. 1989 | Cross- sectional | Riyadh | 1989 | Hemodialysis Bleeding  disorder | High | Health facility | 144 | 122  (84.7%) | 58  (40.3%) | NR | ELISA | High |
|  | Arya et al. 1985 | Cross- sectional | Gizan | 1984 | General population | Low | Health  facility School | 724 | 326  (45.0%) | 286  (39.5%) | 724  (100%) | ELISA | High |
|  | Ashraf et al.  1986 (1) | Cross-  sectional | Gizan | 1984 | General  population | Low | Health  facility | 608 | NR | NR | 608  (100%) | ELISA | Low |
|  | Ashraf et al.  1986 (2) | Cross-  sectional | Gizan | 1985 | Hemodialysis | High | Health  facility | 55 | 52  (94.5%) | NR | 55  (100%) | ELISA | High |
|  | Ayoola et al.  2003 | Retrospective  cohort | Gizan | 1995-1998 | General pop.  Blood donors | Low | Health  facility | 20976 | 20747  (98.9%) | NR | NR | ELISA | Low |
|  | Bajgmoum  2014 ** | Retrospective  cohort | Jeddah | 2000-2010 | HIV | High | Health  facility | 1121 | 1121  (100%) | NR | NR | ELISA | High |
|  | Bakir et al.  1995 | Case-control | Riyadh | 1995 | Healthy  Cancer patients | Low | Health  facility | 221 | 0 (0%) | NR | 221  (100%) | ELISA | High |
|  | Bani et al.  2012 | Cross-  sectional | Jazan | 2012 | Pregnant | Low | Health  facility | 537 | 537  (100%) | 0 (0%) | 496  (92.4%) | Rapid  test | High |
|  | Bashwari et al.  2004 | Cross-  sectional | Dammam | 1998-2001 | Blood donors | Low | Health  Facility | 13443 | 13443  (100%) | NR | 10778  (80.2%) | ELISA | Low |
|  | Eifan et al.  2015 | Cross-  sectional | Al-Kharj | 2011-2013 | General  population | Low | Health  facility | 1128 | 1128  (100%) | NR | NR | ELISA | Low |
|  | Eisa et al. 2012 | Cross-  sectional | Jazan | 2009-2010 | Pre-marital | Low | Health  facility | 28134 | 28134  (100%) | 14073  (50%) | 28134  (100%) | ELISA | Low |
|  | Elbeltagy et al. 2008 | Cross- sectional | Tabuk | 2005-2006 | Blood donors | Low | Health facility | 3192 | 3192  (100%) | 3189  (99.9%) | 3192  (100%) | ELISA | High |
|  | Elbjeirami et  al. 2015 | Retrospective  cohort | Makkah | 2011-2014 | Blood donors | Low | Health  facility | 22963 | 22963  (100%) | NR | NR | CMIA | High |
|  | Elhazmi et al. 1986 | Cross- sectional | Riyadh, Najran, Al- Hafouf, Jaizan,  Khaiber | 1986 | General population | Low | Community | 1336 | 1336  (100%) | 732  (54.8%) | 1336  (100%) | RIA | Low |
|  | Elhazmi 2004 | Retrospective  cohort | Riyadh | 2000-2002 | Blood donors | Low | Health  facility | 24173 | 24173  (100%) | 23583  (97.6%) | 20432  (84.5%) | ELISA | Low |
|  | Ikram et al.  1988 | Cross-  sectional | Madina | 1986-1987 | Blood donors | Low | Health  facility | 369 | 369  (100%) | NR | 234  (63.4%) | ELISA | Low |
|  | Kashgari and Mohamad  1997 | Cross- sectional | Yanbu | 1994 | Healthy workers | Low | Work facility | 332 | 332  (100%) | 332  (100%) | 332  (100%) | ELISA | High |
|  | Khalil et al.  2005 | Cross-  sectional | National | 2005 | Pregnant | Low | Health  facility | 2664 | 2664  (100%) | 0 (0%) | 2664  (100%) | ELISA | High |

|  | Khan and Khan  2001 *** | Retrospective  cohort | Najran | 2001 | Hemodialysis | High | Health  facility | 67 | 67  (100%) | 37  (55.2%) | 53  (79.1%) | NR | High |
| --- | --- | --- | --- | --- | --- | --- | --- | --- | --- | --- | --- | --- | --- |
|  | Mahaba et al.  1997 | Retrospective  cohort | Hail | 1994-1996 | Blood donors  Inpatients | Low  High | Health  facility | 14029 | 12535  (89.4%) | NR | 4560  (32.5%) | ELISA | High |
|  | Mahmoud and  Habib 2012 | Retrospective  cohort | Madina | 2008-2010 | Pregnant | Low | Health  facility | 3060 | 3060  (100%) | 0 (0%) | NR | ELISA | High |
|  | Mansoor et al.  2011 | Cross-  sectional | Riyadh | 2006-2008 | Infertile couples | Low | Health  facility | 924 | 924  (100%) | 462  (50%) | 924  (100%) | ELISA | High |
|  | Mahdi et al.  2000 | Cross-  sectional | Buraidah | 1995-1997 | Blood donors | Low | Health  facility | 11007 | 11007  (100%) | NR | 8003  (72.7%) | ELISA | High |
|  | Mehdi and  Alajlan 2006 | Case-control | Riyadh | 1996-2004 | Non-Hodgkin’s  lymphoma | High | Health  facility | 565 | 565  (100%) | 565  (100%) | NR | ELISA | High |
|  | Njoh 1995 | Retrospective  cohort | Jeddah | 1995 | PWID | High | Health  facility | 1498 | 1498  (100%) | NR | NR | NR | Low |
|  | Panhorta et al.  2005 | Retrospective  cohort | Al-Hafuf | 2000-2003 | Blood donors | Low | Health  facility | 26606 | 26606  (100%) | NR | NR | ELISA | Low |
|  | Parande et al.  1986 | Cross-  sectional | Gizan | 1984-1985 | General  population | Low | Health  facility | 325 | 0 (0%) | 193  (59.4%) | 325  (100%) | ELISA | High |
|  | Ramia et al.  1984 | Cross-  sectional | Riyadh | 1982-1984 | Pregnant | Low | Health  facility | 3020 | 3020  (100%) | 0 (0%) | 3020  (100%) | RPHA | Low |
|  | Sadik et al. 2014 | Retrospective cohort | Taif | 2011 | General pop. Blood donors | Low | Health facility  Community | 1056 | NR | NR | NR | ELISA | Low |
|  | Sarah et al.  2016 | Retrospective  cohort | Hail | 2014-2015 | Blood donors | Low | Health  facility | 361 | 361  (100%) | NR | NR | ELISA | Low |
|  | Shabanah et al.  2019 | Cross-  sectional | Makkah | 2019 | Health care  workers | Medium | Health  facility | 139 | 139  (100%) | 71  (51.1%) | 120  (86.3%) | CMIA | Low |
|  | Shatoor and  Zafer 2002 | Retrospective  cohort | Aseer | 2000-2001 | Blood donors | Low | Health  facility | 5174 | 5174  (100%) | 5174  (100%) | 4664  (90.1%) | ELISA | Low |
|  | Sheth and  Godwin 1986 | Retrospective  cohort | Riyadh | 1983-1984 | Blood donors | Low | Health  facility | 200 | 200  (100%) | 200  (100%) | 200  (100%) | RIA | Low |
|  | Zaini and  Dahlawi 2017 | Retrospective  cohort | Makkah | 2014-2016 | Blood donors | Low | Health  facility | 8820 | 8820  (100%) | NR | 4995  (56.6%) | ELISA | Low |
| **Kuwait** | Almutairi 2013  * | Case-control | Farwaneyah | 2012 | STI and dermatological  conditions | Low High | Health facility | 2446 | 2446  (100%) | NR | NR | NR | Low |
|  | Alnakib et al. 1982 | Case-control | National | 1981 | Blood donors Chronic liver  disease | Low High | Health facility | 198 | 198  (100%) | NR | NR | RIA | Low |
|  | Altawalah et  al. 2015 | Cross-  sectional | Hawali | 2012 | Hemodialysis | High | Health  facility | 1369 | 1369  (100%) | 781  (57.0%) | 744  (54.3%) | ELISA | High |
|  | Altawalah et  al. 2019 | Cross-  sectional | National | 2017 | PWID | High | Health  facility | 521 | 521  (100%) | 489  (93.9%) | NR | ECL | High |
|  | Ameen et al.  2005 | Cross-  sectional | National | 2002 | Blood donors | Low | Health  facility | 12798 | 12798  (100%) | NR | 8561  (66.9%) | CLIA | Low |

| **UAE** | Alamad 2018 | Retrospective  cohort | Sharjah | 2010-2017 | General  population | Low | Health  facility | 372 | 372  (100%) | NR | NR | ECL | High |
| --- | --- | --- | --- | --- | --- | --- | --- | --- | --- | --- | --- | --- | --- |
|  | Alowais et al. 2000 | Cross- sectional | Al-Ain | 1998 | General population | Low | Health facility  College | 8713 | NR | 2439  (28.0%) | NR | ELISA | Low |
|  | Alshaer et al.  2012 | Cross-  sectional | Dubai | 2004-2009 | Blood donors | Low | Health  facility | 169781 | 169781  (100%) | NR | 21997  (13.0%) | ELISA  CMIA | High |
|  | Fakhry et al.  2011 * | Prospective  cohort | Abu Dhabi | 2008-2010 | Police college  applicants | Low | College | 1264 | 1264  (100%) | 1264  (100%) | 1264  (100%) | ELISA | Low |
|  | Trad et al.  2003 | Retrospective  cohort | Abu Dhabi | 1990-2001 | Hemolytic  anemia | High | Health  facility | 16 | 0 (0%) | 4 (25%) | NR | ELISA | Low |
| **Bahrain** | Almahroos and  Ebrahim 1995 | Cross-  sectional | National | 1992 | Hemolytic  anemia | High | Health  facility | 242 | 0 (0%) | NR | NR | ELISA | High |
|  | Janahi 2014 | Cross-  sectional | National | 2000-2010 | General  population | Low | Health  facility | 877892 | NR | NR | NR | ELISA | High |
|  | Yousif et al.  1994 | Cross-  sectional | National | 1994 | HIV | High | Health  facility | 76 | 76  (100%) | NR | NR | ELISA | Low |
| **Oman** | Alawaidy et al.  2013 | Retrospective  cohort | National | 2005 | School children | Low | School | 2065 | 0 (0%) | NR | 2065  (100%) | ELISA | High |
|  | Aldhahry et al. 1994 | Cross- sectional | Muscat | 1993 | Healthy Medical students  Renal disease | Low Medium High | Health facility | 985 | 985  (100%) | NR | NR | ELISA | Low |
|  | Alkindi et al.  2019 | Cross-  sectional | Muscat | 2011-2017 | Hemolytic  anemia | High | Health  facility | 1000 | NR | NR | NR | ELISA | High |
|  | Kaminski et al.  2006 | Retrospective  cohort | Muscat | 2003-2005 | Blood donors | Low | Health  facility | 3290 | 3290  (100%) | NR | NR | CMIA | Low |
|  | Soliman et al 1995 | Retrospective cohort | Muscat | 1995 | Healthy children Sickle cell  anemia | Low High | Health facility | 225 | 0 (0% | NR | NR | Rapid test | Low |
| **Qatar** | Aabdien et al  2020 | Cross-  sectional | National | 2013-2017 | Blood donors | Low | Health  facility | 190509 | 190509  (100%) | NR | NR | CMIA | High |
|  | Almannai and  Riad 2010 * | Retrospective  cohort | Doha | 2003-2004 | Lichen Planus | High | Health  facility | 100 | 100  (100%) | NR | NR | ELISA | Low |
|  | Hamilton et al.  2010 | Cross-  sectional | Doha | 2008-2009 | Athletes | Low | Health  facility | 780 | 780  (100%) | 780  (100%) | 455  (58.3%) | ELISA | High |
|  | Rikabi et al.  2009 | Retrospective  cohort | National | 2000-2005 | Liver disease | High | Health  facility | 915 | 915  (100%) | NR | NR | NR | Low |
| **Multi- national** | Alawaidy et al. 2006 | Cross- sectional | Oman Qatar  UAE | 2000 | Pregnancy | Low | Health facility | 1694 | 1694  (100%) | 0 (0%) | NR | ELISA | High |
|  | Almawi et al.  2004 | Cross-  sectional | KSA  Bahrain | 2004 | Blood donors  Hemodialysis | Low  High | Health  facility | 10159 | 10159  (100%) | NR | NR | NR | Low |

* Abstract

** Thesis

*** Letter to the editor

CLIA: Chemiluminescent Immunoassays

CMIA: Chemiluminescent Microparticle Immunoassays ECL: Electrogenerated Chemiluminescence Immunoassay ELISA: Enzyme-linked Immunosorbent Assay

HIV: Human Immunodeficiency Virus NR: Not reported

KSA: Kingdom of Saudi Arabia PWID: Person Who Inject Drugs RIA: Radioimmunoassay

RPHA:Reverse phase passive haemagglutination STI: Sexually transmitted Illness

UAE: United Arab Emirates

**Appendix D. Forrest plot for the overall HBV prevalence in the GCC region**

**Appendix E1:** Quality assessment of cross-sectional studies


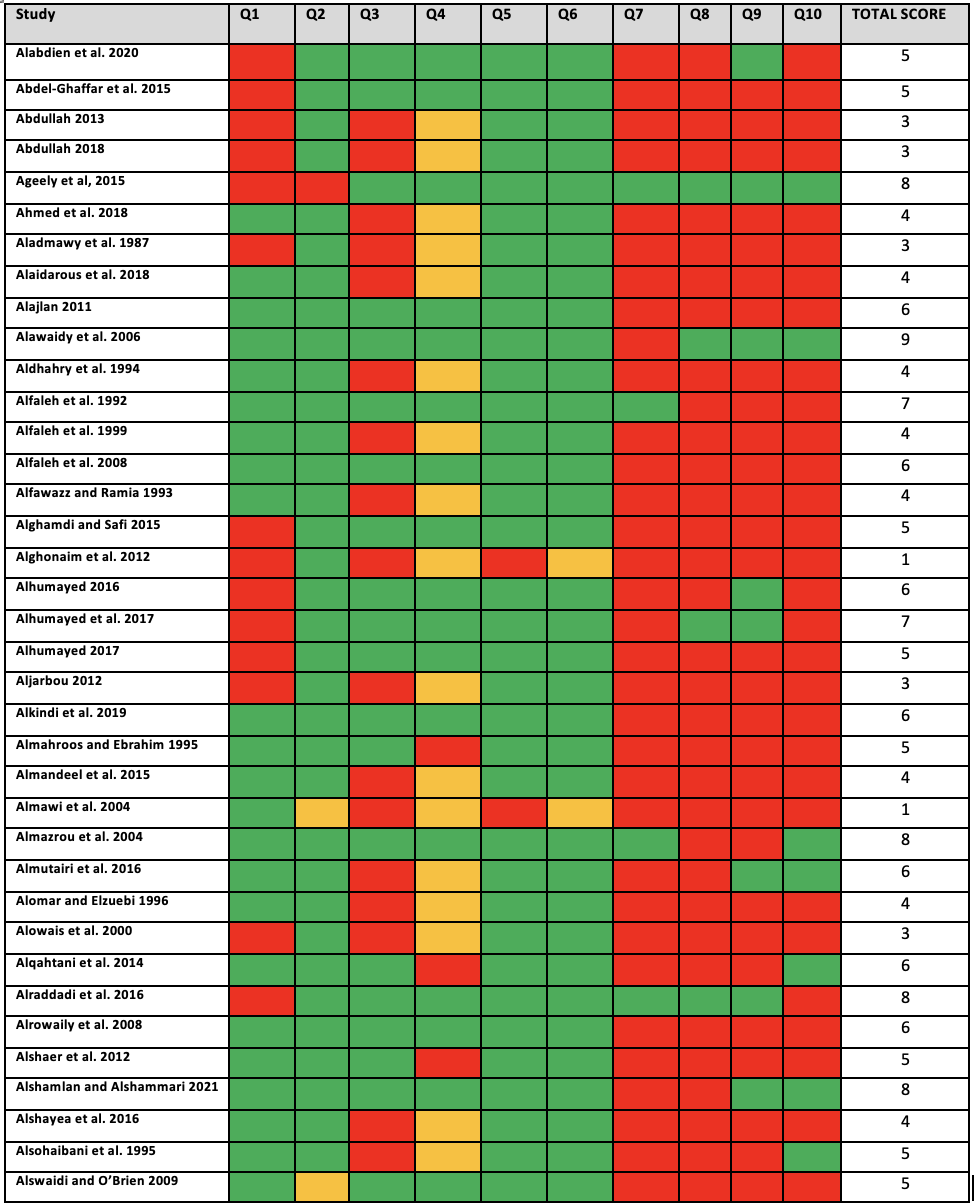


continued…

Appendix E1 continued


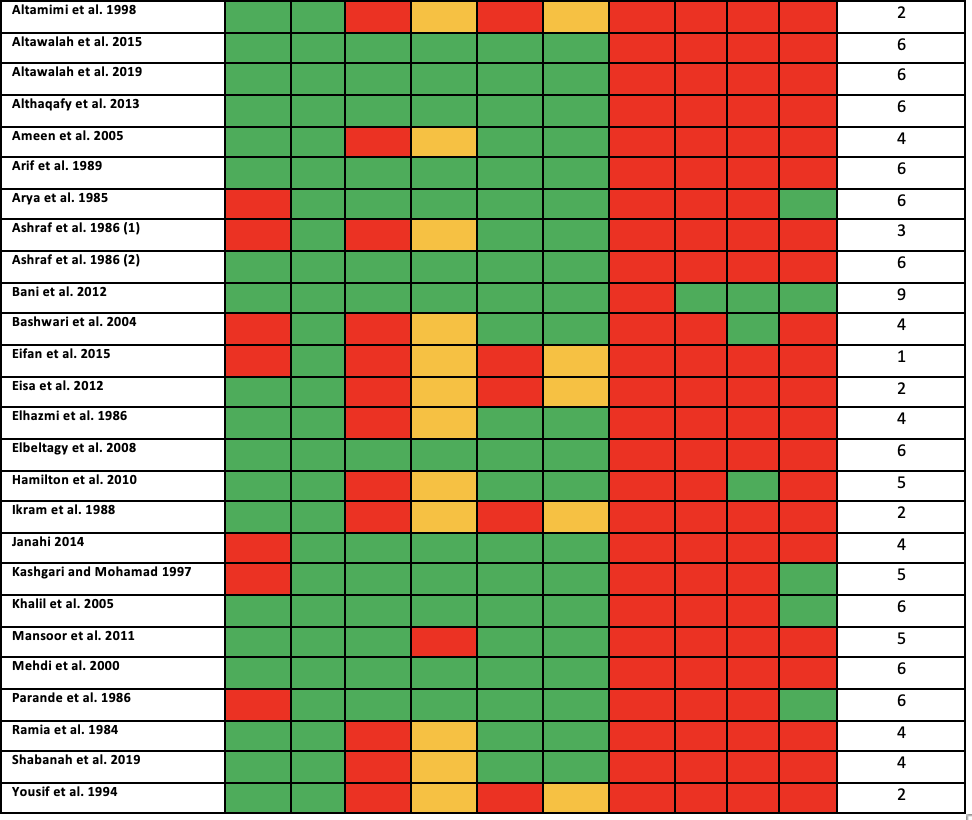


**Appendix E2:** Quality assessment of cohort studies


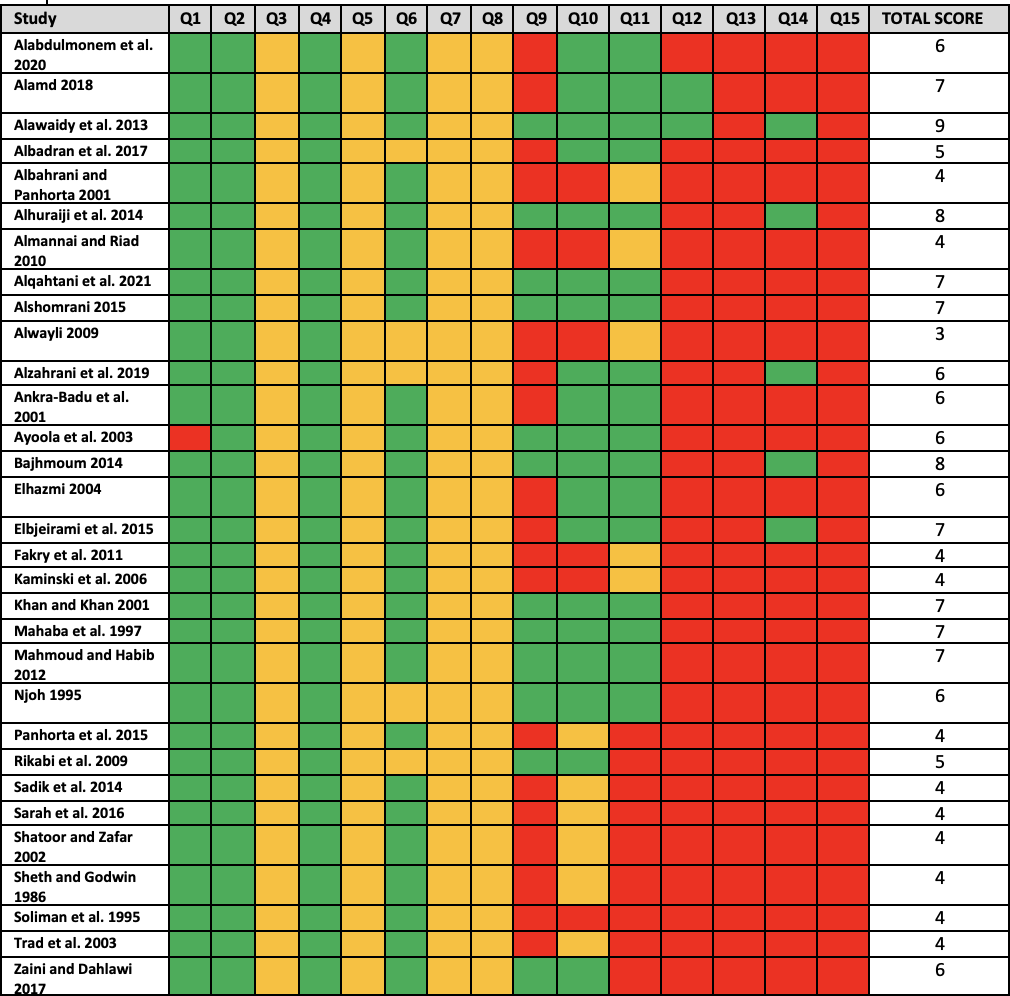


**Appendix E3:** Quality assessment of case-control studies


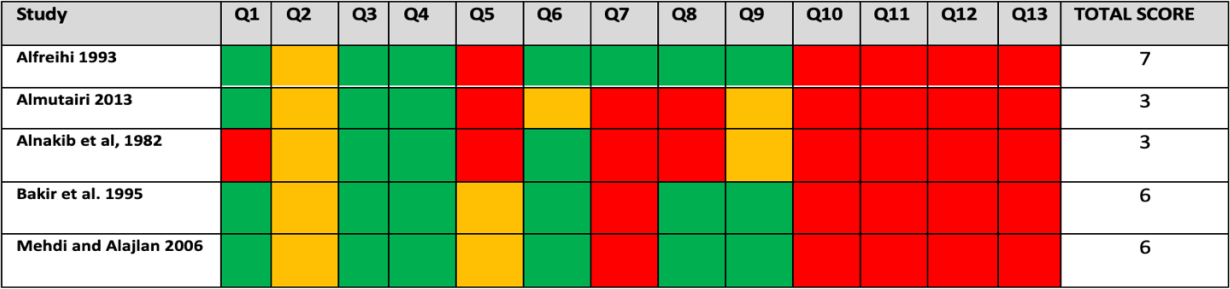


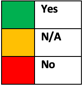


***Key for quality assessment**
